# Book Review

**DOI:** 10.1155/1998/54697

**Published:** 1998


HPB Surgery, 1998, Vol. 10, pp. 403–404
Reprints available directly from the publisher
Photocopying permitted by license only

© 1998 OPA (Overseas Publishers Association)
Amsterdam B.V. Published under license
under the Harwood Academic Publishers imprint,
part of The Gordon and Breach Publishing Group.
Printed in India.

### Book Review

**ART SURGERY AND TRANSPLANTATION, Edited by Ann Lenehan and Jane Smith, Published by Williams & Wilkins Europe Limited, 227 pp., £45.00,(ISBN 0-683-23094-8), 193 width × 265 length, 186 illustrations, Available at Waverly Europe Limited (Tel: 0171 385 2357/Fax: 0171 385 2922).**

This book by Sir Roy Calne, probably the major influence in British transplantation, is a fascinating review of the association between art and medicine, artists and doctors and a history of transplantation as an acceptable treatment modality. There could be few people practising transplantation with such first hand knowledge and experience to produce such a book. In addition his interest in drawing and painting gives further fascination to this volume which I suspect is primarily a vehicle for his own paintings.

In the introduction he discusses the emotions of transplantation, with which many of us within the field will empathise, and tells how, through one of his liver transplantees, a renown artist, his life long interest in art was rekindled.

In the first few chapters he discusses the evolution of painting and its use in communication, his own urge to paint and the use of drawing and sketching in the life of a professor of surgery. He compares "the similarity of painting and drawing to surgery and research, both requiring careful planning, skill, technique and familiarity with tools. However a poor painting can be discarded whereas poor surgery leads to patient harm."

He next discusses man's appreciation of the painted image and the influence of the renaissance on the evolution of painting and art releasing it from its previous religous restraints and then points out the images of anatomy and sickness used by many famous artists. He then gives examples of artists who had undergone medical training with samples of their work.

The rest of the book is concerned with transplantation and discusses his own artistic interest of painting operations, patients and colleagues. There is a long chapter on the short history of modern transplantation with reference to the major contributors to the speciality as we know it, including the surgical procedure, the science of immunology, tissue typing, immunosuppressive therapy including current and future developments. This chapter concludes with a section on donors, live and cadaveric and the possibility of animal organs (xenografts) in the future.

The final three chapters deal with the relationship of patients, doctors, nurses and scientists involved in transplantation, the greatly improved quality of life following engraftment although there may be complications of the surgery and immunosuppressive therapy and finally the ethics of transplantation.

The whole of the book is extensively illustrated with medical paintings and illustrations the majority of which are Sir Roy's own works. The appreciation of art is very much in the eye of the beholder and I must admit many of Sir Roy's works I do not personally like. However his use of colour does a great deal to portray the sickness fear and apprehension of his patients and many of his sketches I do like very much. I do like his ideas of sketching patients particularly children and then getting them to colour the drawings perhaps it is an idea many of us could use to foster doctor patient relationships.

Despite my own personal reservations I know there are many who do like Sir Roy's work and I feel this is an interesting book to anyone who is involved in transplantation and would make a valuable addition to any library.

PETER GRIFFIN

